# Edible liquid marbles stabilized with millimeter-sized spherical particles

**DOI:** 10.1016/j.crfs.2024.100899

**Published:** 2024-11-09

**Authors:** Diagne Mame-Khady, Takanori Yasui, Shota Sugiyama, Anne-Laure Fameau, Tomoyasu Hirai, Yoshinobu Nakamura, Syuji Fujii

**Affiliations:** a Toulouse INP-ENSIACET, 4, Allée Émile Monso, CS 44362, 31030, TOULOUSE, CEDEX 4, France; b Division of Applied Chemistry, Environmental and Biomedical Engineering, Graduate School of Engineering, Osaka Institute of Technology, 5-16-1 Omiya, Asahi-ku, Osaka, 535-8585, Japan; c Université Lille, CNRS, INRAE, Centrale Lille, UMR 8207 - UMET - Unité Matériaux et Transformations, F-59000, Lille, France; d Department of Applied Chemistry, Faculty of Engineering, Osaka Institute of Technology, 5-16-1 Omiya, Asahi-ku, Osaka, 535-8585, Japan; e Nanomaterials Microdevices Research Center, Osaka Institute of Technology, 5-16-1 Omiya, Asahi-ku, Osaka, 535-8585, Japan

**Keywords:** Liquid marble, Edible particle, Silver dragees, Fatty acid, Shape design

## Abstract

Liquid marbles (LMs) are millimeter-sized liquid droplets in a gaseous phase coated with solid particles. The LM technology allows liquid droplets to be treated as solid particles. As an LM stabilizer, edible particles are of particular interest, especially for applications in the food industry. However and surprisingly, there are limited numbers of reports describing LMs stabilized with edible particles. In this study, we utilize silver dragees, which are millimeter-sized spherical sugar beads coated by silver overlayers used for decorating cakes and cookies, as edible particles to stabilize LMs. The silver dragees surface was modified using stearic acid to work as an effective LM stabilizer by adsorbing at the gas-liquid interface. LMs with millimeter and centimeter sizes can be fabricated. Due to high adsorption energy and jamming effect of the modified dragees at gas-liquid interface, LMs with non-equilibrium shapes including intricate ones with varying curvatures can be prepared. Developing LMs with customizable shapes is crucial for expanding their potential applications and versatility as functional systems for food applications. We therefore show how these customizable-shaped LMs can be utilized as an edible decoration material for food applications.

## Highlights

• Liquid marbles are droplets coated with solid particles in a gaseous phase.
• Limited reports exist on liquid marbles stabilized with edible particles.
• Silver dragees, sugar beads with silver coats, are used to stabilize liquid marbles.
• Hydrophobically modified dragees enable creating customizable liquid marbles.
• These liquid marbles can be used for food applications.

## Graphical abstract

This study presents a new approach to creating edible liquid marbles. The silver dragees were hydrophobized by stearic acid enabling them to adhere to the air-liquid interface and serve as an effective stabilizer. The customizable properties of these liquid marbles showcase an innovative potential in food science applications.

**Figure:**
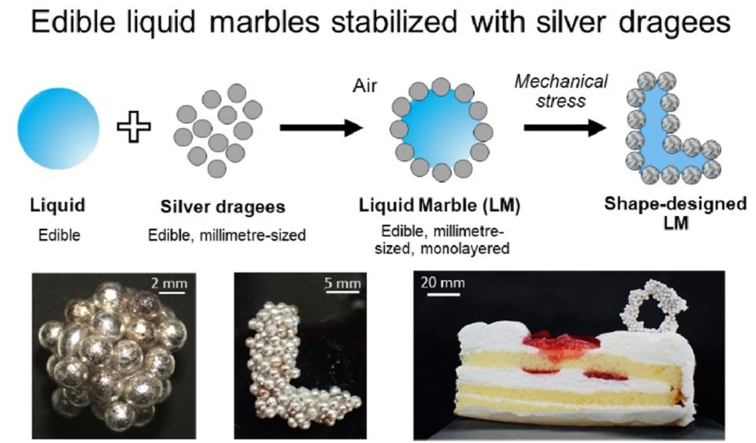

## 1 Introduction

In recent years, a significant trend in food science has emerged concerning the use of various types of solid particles to stabilize oil-water, air-water and air-oil interfaces (Dickinson, 2020). Food scientists have long understood the importance of fat crystals as solid particles in managing the stability and texture of edible water-in-oil emulsions and air-in-oil foams (Rousseau, 2013; Fameau et al., 2017). However, it is only recently that the significant potential of other type of solid particles as stabilizers for edible oil-in-water emulsions and air-in-water and air-in-oil foams has gained widespread recognition (Lam et al., 2014; Linke and Drusch, 2018; Murray, 2019; Jiang et al., 2021; Niroula et al., 2021; Cui et al., 2021; Del et al., 2024; Guida et al., 2024). Foams and emulsions are very well known soft matter systems for food applications. In 2001, a new soft matter system called liquid marble (LM) was described for the first time (Aussillous and Quéré, 2001). LM is a millimeter-sized liquid droplet covered and stabilized by solid particles in a gaseous phase (Aussillous and Quéré, 2006; McHale and Newton, 2015; Fujii et al., 2016a; Bormashenko, 2017; Oliveira et al., 2017; Tenjimbayashi et al., 2023). The solid particles create a non-wetting shell around the liquid droplet, and the resulting LM can be treated as solid system with a liquid core. The protective layer of solid particles on the droplet surface prevents the liquid core from touching the supporting substrates. Consequently, this shielding effect protects the inner liquid from external contamination and gives LMs their non-wetting and non-sticking properties. Such LMs have recently gathered significant interest due to their ability to encapsulate functional materials. This capability has led to various applications, including use as miniature reactors (Sato et al., 2015; Koh et al., 2017; Salehabad et al., 2019), sensors (Dupin et al., 2009; Bormashenko and Musin, 2009; Fujii et al., 2010; Tian et al., 2010; Adamatzky et al., 2020), carriers of materials (Paven et al., 2016; Oaki and Fujii, 2024), accelerometers (Zeng and Zhao, 2010), and pressure-sensitive adhesives (Fujii et al., 2016b).

Synthetic organic, inorganic, and hybrid particles, as well as natural particles, have been shown to work as a stabilizer for LMs (Aussillous and Quéré, 2001; McHale and Newton, 2015; Fujii et al., 2016a; Pike et al., 2002; Kasahara et al., 2019). Among these, edible particles are of particular interest as LM stabilizers, especially for applications in the food industry. However and surprisingly, there have been limited numbers of reports describing the LMs stabilized with edible particles (Salehabad et al., 2019; Kawamura et al., 2012; Wang et al., 2021). Until now, most studies on LMs have used solid particles with diameters ranging from a few nanometers to a few tens of micrometers, or particle aggregates with undefined sizes and shapes. Yet, there have been a limited number of studies that use millimeter-sized particles that are monodispersed in size and shape to stabilize LMs (Geyer et al., 2019). One advantage of using millimeter-sized particles is that the resulting LM can be easily observed by the naked eye which can be important for food applications in terms of visual aspect, but also in terms of sensorial properties.

Moreover, in the food industry, the phase change technology from liquid state to solid state is important and has been applied in preparations of jellies, chocolates, mousses, etc. Solid foods have been shown to trigger stronger appetite and dietary responses than liquid foods (Leathwood and Pollet, 1988; Mattes and Rothacker, 2001). In addition, the sensation of satiety occurs more quickly after consuming solid foods in contrast with liquid ones. This sensation lasts longer, delaying the return of hunger (Leathwood and Pollet, 1988). Therefore, there has been increasing interest in developing technology to solidify comestible liquids, and LMs could be an interesting new technology for this object in food science.

**Fig. 1:**
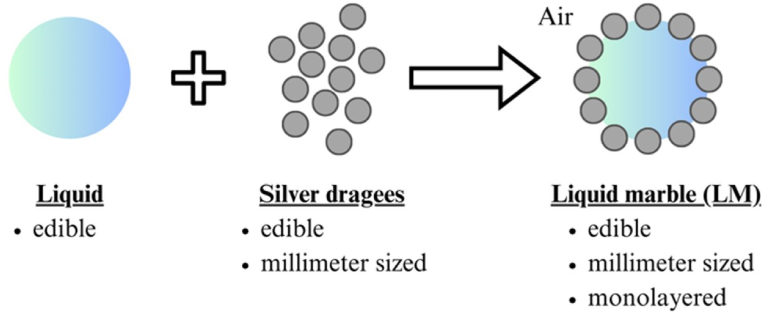
Fabrication of edible liquid marble (LM) stabilized with millimeter-sized silver dragees adsorbed at air-liquid interface.

In this communication, we proposed the utilization of millimeter-sized silver dragees, which are spherical sugar beads coated by silver overlayers used for decorating cakes and cookies, as an LM stabilizer (Fig. 1). Hydrophobization of silver dragees was first conducted using an edible hydrophobizer, stearic acid (SA), to change the wetting behavior of the particles and make them suitable to fabricate stable LMs. Then, we investigated the stabilization and shape design of LMs using the hydrophobic silver dragees as an LM stabilizer. The formability and microstructures of the LMs were extensively characterized using the naked eye and a stereo microscope. Developing LMs with customizable shapes is crucial for expanding their potential applications and versatility as food systems, that is why different shapes were produced and studied.

## 2 Materials and methods

### 2.1 Materials

All chemicals were purchased as reagent grade and used as obtained unless otherwise stated. Silver dragees were obtained from Pioneer Kikaku Co. (Kanagawa, Japan). Stearic acid (SA, >98.0%), and behenic acid (>80%), were obtained from Tokyo Chemical Industry Co., Ltd. (Tokyo, Japan). Palmitic acids was obtained from FUJIFILM Wako Pure Chemical Corporation. Polyglycerin (PGL, #750, molecular weight, 750; viscosity, 5.80 ± 0.17 Pa s; surface tension, 48.9 ± 1.3 mN m^−1^) and polyglyceryl monolaurate (ML-750, molecular weight, 940; viscosity, 62 Pa s; surface tension, 16.7 ± 0.1 mN m^−1^) were kindly donated from Sakamoto Yakuhin Kogyo Co., Ltd. (Osaka, Japan). Ethanol (95%) was obtained from Sigma-Aldrich (Saint-Louis, Missouri, USA). Honey was obtained from Saitama Yoho Co., Ltd. (Saitama, Japan), and bought at “Life” supermarket (Osaka, Japan).

### 2.2 Silver dragees modification protocol by fatty acid

Hydrophobization was conducted as follows: SA (0.5 g) was dissolved in ethanol (100 mL) at 25 °C with handshaking (5.0 g/L). To this ethanol solution of SA, the pristine silver dragees (25 g) were introduced and then dispersed. The dispersion was left to stand for 1 h with hand mixing every 10 min. The resulting silver dragees were purified via decantation-based washing using ethanol (3 times), followed by vacuum drying using a freeze dryer (FDU-1200, Tokyo Rikakikai Co., Ltd.) at < 87.6 Pa. The hydrophobization of silver dragees were conducted using other fatty acids, namely palmitic acid and behenic acid in the same manner: Note that the behenic acid was heated up to 50 °C to dissolve in ethanol due to its low solubility at room temperature (Fameau et al., 2021).

### 2.3 Observation with a stereoscopic microscope

The silver dragees were observed from the top using a stereoscopic microscope (STZ-161-TLED-1080 M, Shimadzu Rika Co., Tokyo, Japan) and a digital imaging system (Moticam1080BMH, Shimadzu Rika Co., Tokyo, Japan). The contact angles of the silver dragees at air-liquid interfaces were estimated using the stereomicroscopy images (Fujii et al., 2013, 2018). The resulting data are presented as mean diameter ± standard deviation.

### 2.4 Observation with an optical camera

The LMs stabilized with silver dragees were observed from the top and side using a digital camera (Tough TG-6, Olympus Co., Tokyo, Japan).

### 2.5 Scanning electron microscopy (SEM)

SEM studies were conducted using HITACHI, TM4000 Miniscope II operating at 15 kV with Au sputter-coated (Elionix SC-701 Quick Coater, Tokyo, Japan) dried samples.

## 3 Results and discussion

### 3.1 Particles modification and liquid marbles production

Silver dragees, also known as sugar pearls, are millimeter-sized spherical sugar particles coated by silver overlayers, generally used to decorate cookies, cakes, and other pastries. The number-average particle diameter (*D*_n_) of the pristine silver dragees was 1.90 ± 0.11 mm, confirmed by the stereomicroscope (Figs. S1a and b). The thickness of the silver overlayer was 0.32 ± 0.06 μm, which was measured from SEM image of silver dragees obtained after removing of sugar core component using water. The volume ratio of sugar core/silver shell was calculated to be 99.92/0.08. We investigated the applicability of the silver dragees as an LM stabilizer. As an internal potential edible model liquid of the LMs, we first utilized PGL. Generally, water is utilized as an inner liquid of the LMs. Unfortunately, the silver dragees could be dissolved in water and could not stabilize LMs. The pristine silver dragees were relatively hydrophilic; the dragees formed a contact angle at the air-PGL interface (*θ*, measured through the PGL phase) of 65 ± 7°, which was determined by direct observation using a stereomicroscope (Fig. S1c). As a result, pristine dragees could not stabilize stable LMs, and dragees aggregates where PGL functioned as an adhesive were formed rather than LMs.

**Fig. 2:**
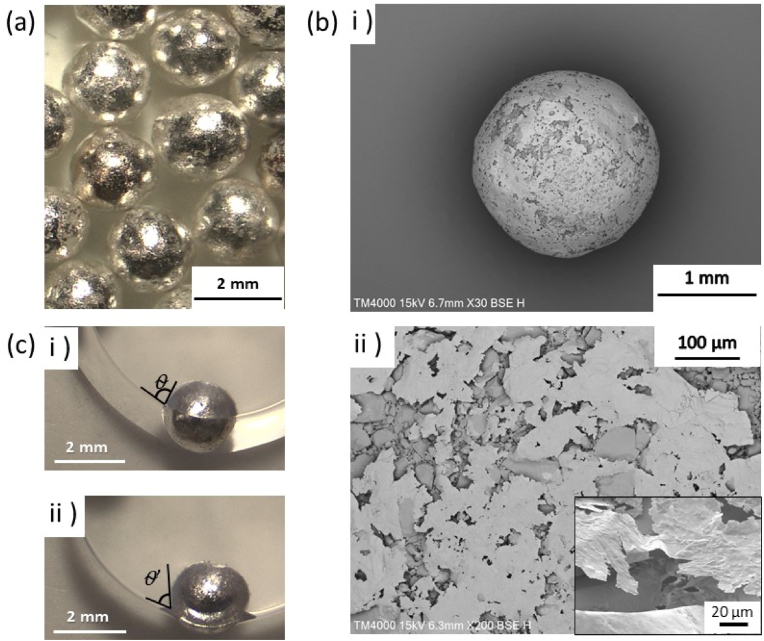
(a) Stereomicroscopy and (b) SEM images of steaic acid (SA)-modified silver dragees (SA-silver dragees), ii) Magnified image of Fig. 2b-i). An insert of Fig. 2b-ii) is the silver shell after removing the sugar core of the SA-silver dragees using water. (c) SA-silver dragees adsorbed at air-polyglycerin (PGL) interface: i) before and ii) after application of mechanical stress.

To reach a suitable contact angle for LMs formation, hydrophobization of the silver dragees was required. Here, we utilized SA as a biocompatible and biodegradable hydrophobizer (Qiu et al., 2020; Fameau and Marangoni, 2022; Tyowua et al., 2022). Carboxyl groups are known to attract with silver surface (Jadhav, 2011), and therefore SA can adsorb to the surface of silver dragees. We developed a simple hydrophobization protocol leading to surface modification of the silver dragees. Visual observations confirmed that the silver dragees did not flocculate during and after the surface modification process. Optical microscopy studies after vacuum drying confirmed that the SA-modified silver dragees (SA-silver dragees) maintained their spherical shape and the diameter (*D*_n_) was estimated to be 1.87 ± 0.12 mm (Fig. 2a). Importantly, the *D*_n_ did not change significantly after hydrophobization. The surface of the SA-silver dragees shows a several tens micrometer-sized surface roughness and the thickness of the silver overlayer was estimated to be 0.31 ± 0.08 μm, which was almost identical to that before the hydrophobization (Fig. 2b–ii). LMs can be successfully fabricated by rolling PGL droplets (approximately 30 μL) over a layer of SA-silver dragees **(Movie 1)**. The *θ* value at the air-PGL interface for the SA-modified dragees was found to be 80 ± 3° (Fig. 2c–i), which was high enough to stabilize LMs (Asaumi et al., 2020).

Supplementary video related to this article can be found at https://doi.org/10.1016/j.crfs.2024.100899

The following is/are the supplementary data related to this article:

Movie S1 Movie S1

### 3.2 Liquid marbles characterization and properties

**Fig. 3:**
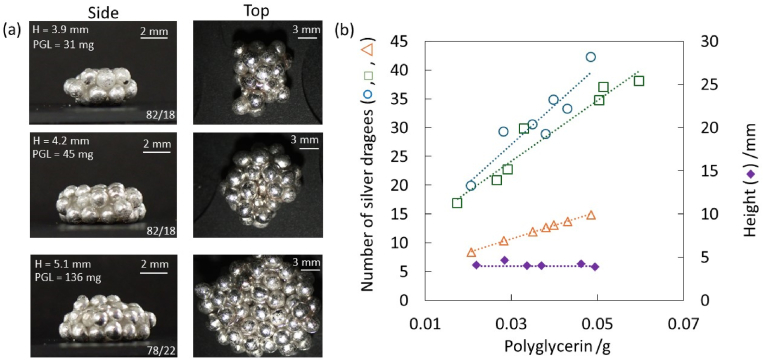
(a) Optical photography of liquid marbles containing PGL as inner liquid stabilized with SA-silver dragees. The height (H), the amount of PGL used (PGL) and the dragees/liquid weight ratio (bottom right corner) are shown in the image. (b) Comparison of the number of silver dragees adsorbed to one LM: () Pristine silver dragees (), SA-silver dragees (), theoretical values calculated assuming a spherical droplet shape, and () height of liquid marbles depending on the amount of PGL used. Dotted lines are the trendlines of each data series.

Stereomicroscopy observation confirmed the formation of non-spherical LMs using PGL with various volumes (Fig. 3a a nd S2). The LM exhibited noticeable surface roughness, with the roughness directly corresponding to the size of the silver dragees (Fig. 3a). This roughness is a result of the dragees' physical presence at the air-liquid interface, creating a textured surface pattern based on their dimensions. No matter the possible amount of liquid used, the weight ratio of the LM remains around 80% SA-silver dragees and 20% liquid, whereas it is around 90% and 10% for pristine dragees. The larger LM could be fabricated using PGL droplet (∼100 μL) and the shape was oblate because of the gravity effect (Fig. 3a and S2). The number of silver dragees adsorbed to the LM was determined for LMs containing various liquid volumes by gravimetric analysis. The numbers were higher than the theoretical calculations, based on the assumption of a monolayer of particles adhering to a perfectly spherical droplet in a close-packed manner (Fig. 3b). The increased number of particles observed experimentally can be attributed to the LM deviating from a spherical shape, thereby increasing its surface area. The numbers of silver dragees determined for aggregates prepared using pristine silver dragees were larger than those measured for LMs stabilized with SA-silver dragees. This should be due to the incorporation of silver dragees into the liquid phase. Without the surface modification, the silver dragees are adsorbed at the air-PGL interface and absorbed into the PGL liquid phase. The surface modification reduces the amount of absorption.

Regarding the height of the marbles, they remain around the same value and fluctuate by a few millimeters (Fig. 3b). The deviations from spherical dimensions were explained by the relationship between the radius (*R*_0_) of the quasi-spherical PGL droplet and the capillary length (*κ*^−1^) (see Equations (Equation 1), (Equation 2))).

(Equation 1) $$R_{0}={\left(\frac{3V}{4\pi }\right)}^{\frac{1}{3}}$$

(Equation 2) $$\kappa^{-1}=\sqrt{\frac{\gamma }{\rho \;g}}$$

*V* represents the volume of the PGL droplet. When $R_{0}\ll \kappa^{-1}$, the surface tension dominates over gravity resulting in a quasi-spherical droplet. Conversely, when $R_{0}\gg \kappa^{-1}$, gravity predominates over the surface tension, the droplet resembles a puddle. With a surface tension (*γ)* value of 48.9 mNm^−1^ for the PGL, *κ*^−1^ was estimated as 1.9 mm. This *κ*^−1^ value suggests that LMs with *R*_0_ < 1,9 mm should adopt near-spherical shapes and those with *R*_0_ > 1.9 mm assume puddle-like shapes. Considering the diameter of silver dragee is 1.87 mm, the height of LMs could not exceed >5 mm (Hayashi et al., 2024), and the formation of near-spherical LMs is unlikely.

The adsorption energy of the silver dragees at the air-PGL interface was determined using the contact angle of 80° to be – 9.04 × 10^13^ *kT* based on the following equation (Levine et al., 1989).

(Equation 3) Δ*G* = - *γ*_al_*πR*_p_^2^ (1 - cos*θ*)^2^

Here *γ*_al_ is the surface tension of PGL (48.9 ± 1.3 mN m^−1^) and *R*_p_ is the radius of the SA-silver dragees. The adsorption energy per one dragee is substantial enough that the adsorption of dragees at the air-PGL interface becomes irreversible at ambient temperature. (Note that adsorption energies of molecular-level surfactants are several *kT* - several tens of *kT* (Danov and Kralchevsky, 2012), and they show reversible adsorption at the interfaces.) These results shown above confirmed surface modification of the silver dragees with the SA is essential for the fabrication of LMs. The negative sign implies a spontaneous absorption. Even with their considerable mass, the SA-silver dragees naturally adhere to the air-PGL interface of a droplet, aiming to minimize the interfacial free energy.

### 3.3 Versatility of liquid marbles and possible applications

**Fig. 4:**
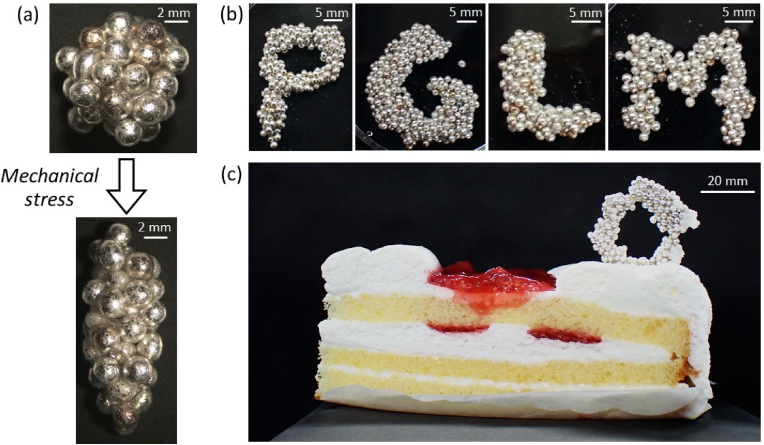
(a) Stereomicroscopy image of a liquid marble (PGL as inner liquid) before and after the application of mechanical stress. (b) Digital camera images of liquid marbles (PGL as inner liquid) shaped as letters. (c) Digital camera image of a liquid marble (honey as inner liquid) on top of a cake.

Due to this extremely high adsorption energy, the LMs stabilized with the millimeter-sized silver dragees showed a remarkable ability to have customizable shapes. The jamming of solid particles at air-liquid interfaces makes them inelastic and could stabilize their shapes (Subramaniam et al., 2005; Stratford et al., 2005; Cui et al., 2013). The steric jamming effect on particles at interfaces has realized non-spherical LMs (Geyer et al., 2019). The silver dragees adsorbed at the air-PGL interface create an inelastic and plastically deformable surface (in other word, when external forces are applied, the surface will change shape and remain in that altered state rather than bouncing back to its original form), enabling the design of various LM shapes by application of mechanical stress (Fig. 4a). It should be noted that internal PGL sometimes leaks out from the LMs during the shape control. The optical microscopy studies confirmed that the contact angle of SA-silver dragees at the air-PGL interface decreased from 80 ± 3° to 57 ± 8° by application of shear stress, indicating position change of the dragees at the interface (Fig. 2c-ii and 4a). These results should suggest that the SA-silver dragees adsorbed at the air-PGL interface in a meta-stable Cassie-Baxter state due to the surface roughness of the dragees and depinning occurred by the application of mechanical stress (Aono et al., 2022). Fig. 4b illustrates letter-shaped LMs formed by merging and/or applying mechanical stress on LMs stabilized with the SA-silver dragees.

We were able to fabricate LMs using SA-silver dragees hydrophobized with SA at lower and higher concentrations (2.5 g/L and 7.5 g/L) and observed contact angles of 91 ± 5° and 96 ± 6° for SA concentration systems of 2.5 g/L and 7.5 g/L, respectively (Figs. S3 and S4). We also used other long chain fatty acids to show the versatility of our approach to hydrophobized the silver dragees. We showed that palmitic acid and behenic acid could also work as an LM stabilizer (Figs. S3 and S4). The optical microscopy observations revealed contact angles of 78 ± 7° for the palmitic acid, and 96 ± 5° for the behenic acid.

Furthermore, it is possible to utilize other edible liquids as the inner liquid of the LM. Figs. S5 and S6 show that using high-viscosity edible liquids such as honey and polyglyceryl monolaurate still allows LM creation. The optical microscopy studies displayed a contact angle of SA-silver dragees at an air-honey interface of 108 ± 6°. This value decreased to 94 ± 1° with the application of mechanical stress (Fig. S7). This higher contact angle compared to that observed with PGL suggests that the viscosity of the internal liquid can influence the stability of the LM.

Finally, we show the possible application of the edible LMs for molecular gastronomy. After the transformation of the LMs containing honey into the desired shape, the water component in the honey is allowed to evaporate (water content of the honey, 17 wt%), resulting in solidification of the internal liquid phase. The dried LMs can free stand and could be placed on a dessert, such as a cake, leading to new sensorial characters based on the visual appearance and texture of LMs (Fig. 4c).

## 4 Conclusion

In this study, we have shown how the silver dragees were effectively hydrophobized using SA, allowing them to adhere to the air-liquid interface and function as an efficient stabilizer for LMs. The high adsorption energy of the dragees ensured their retention at the interface, resulting in interfacial jamming. This phenomenon led to LM's formation with non-equilibrium shapes including intricate ones with varying curvatures. The edible LMs developed in this study show significant potential for various applications, especially in food science, owing to their unique properties, including customizable visual appearance and shape.

## CRediT authorship contribution statement

**Diagne Mame-Khady:** Methodology, Investigation, Writing – original draft. **Takanori Yasui:** Methodology. **Shota Sugiyama:** Methodology. **Anne-Laure Fameau:** Investigation, Writing – review & editing. **Tomoyasu Hirai:** Methodology. **Yoshinobu Nakamura:** Methodology. **Syuji Fujii:** Conceptualization, Methodology, Investigation, Writing – original draft, Writing – review & editing, Supervision, Project administration, Funding acquisition.

## Declaration of competing interest

The authors declare that they have no known competing financial interests or personal relationships that could have appeared to influence the work reported in this paper.

## Data Availability

Data will be made available on request.

## Appendix A Supplementary data

Supplementary data are available free of charge via the Internet. Details on the characterization method of the silver dragees and LMs using optical photography, stereomicroscopy, SEM, and contact angle measurement.
