# Supplementary material for: Edible liquid marbles stabilized with millimeter-sized spherical particles
Source: Curr Res Food Sci. 2024 Nov 9;9:100899. doi: 10.1016/j.crfs.2024.100899 (PMC11607657; doi:10.1016/j.crfs.2024.100899)
Supplement: Supplementary file 2 — Supplementary data are available free of charge via the Internet. Details on the characterization method of the silver dragees and LMs using optical photography, stereomicroscopy, SEM, and contact angle measurement. [file mmc1.docx]

**Supporting information**

**Edible liquid marbles stabilized with**

**millimeter-sized spherical particles**

Diagne Mame-Khady^1^, Takanori Yasui^2^, Shota Sugiyama^2^, Anne-Laure Fameau^3^*,

Tomoyasu Hirai^4,5^, Yoshinobu Nakamura^4,5^, Syuji Fujii^4,5^*

*^1^Toulouse INP-ENSIACET*

*4, Allée Émile Monso - CS 44362 - 31030 TOULOUSE CEDEX 4 - France*

*^2^Division of Applied Chemistry, Environmental and Biomedical Engineering,*

*Graduate School of Engineering*

*Osaka Institute of Technology, 5-16-1 Omiya, Asahi-ku Osaka 535-8585, Japan.*

*^3^Université Lille, CNRS, INRAE, Centrale Lille, UMR 8207 - UMET - Unité Matériaux et Transformations, F-59000 Lille, France.*

*^4^Department of Applied Chemistry, Faculty of Engineering,*

*Osaka Institute of Technology, 5-16-1 Omiya, Asahi-ku Osaka 535-8585, Japan.*

*^5^Nanomaterials Microdevices Research Center*

*Osaka Institute of Technology, 5-16-1 Omiya, Asahi-ku Osaka 535-8585, Japan.*

* Author to whom correspondence should be addressed

syuji.fujii@oit.ac.jp

Number of pages in the Supporting Information: 5 (page S1 to page S5)

Number of Figures in the Supporting Information: 7 (Figure S1 to Figure S7)

Number of Videos in the Supporting Information: 1


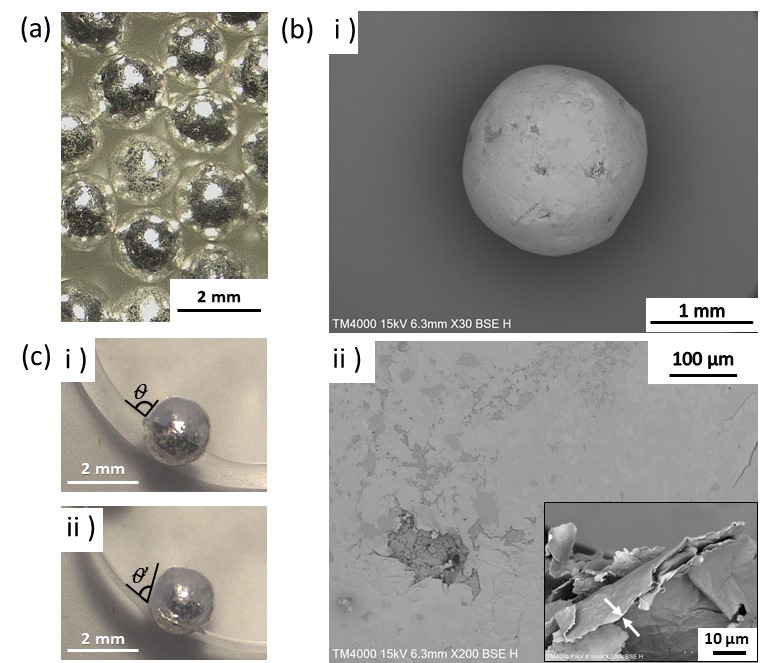


**Figure S1:** (a) Stereomicroscopy and (b) SEM images of silver dragees, ii) Magnified image of S1b.i). An insert of Fig.S1b.ii) is the silver shell after removing the sugar core of the silver dragees using water. The arrows indicate the thickness of silver shell. (c) Silver dragees adsorbed at air-PGL interface i) before and ii) after application of mechanical stress.


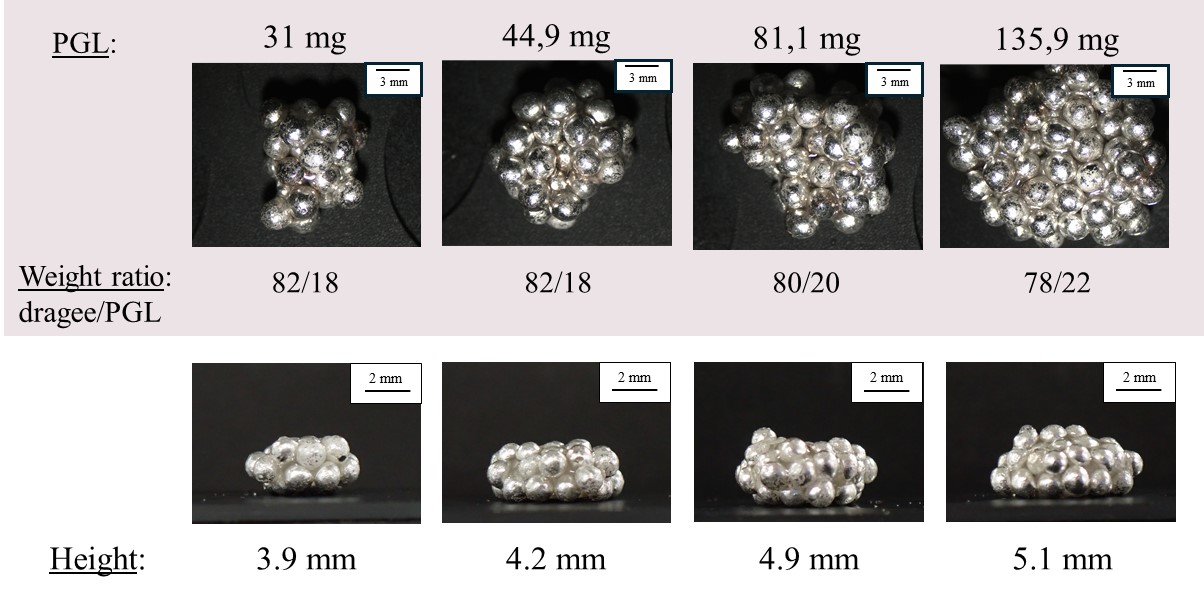


**Figure S2:** Stereomicroscopy images of liquid marbles containing PGL as an inner liquid stabilized with SA-silver dragees. The height, the amount of PGL used and the dragees/PGL weight ratio are shown in the image.


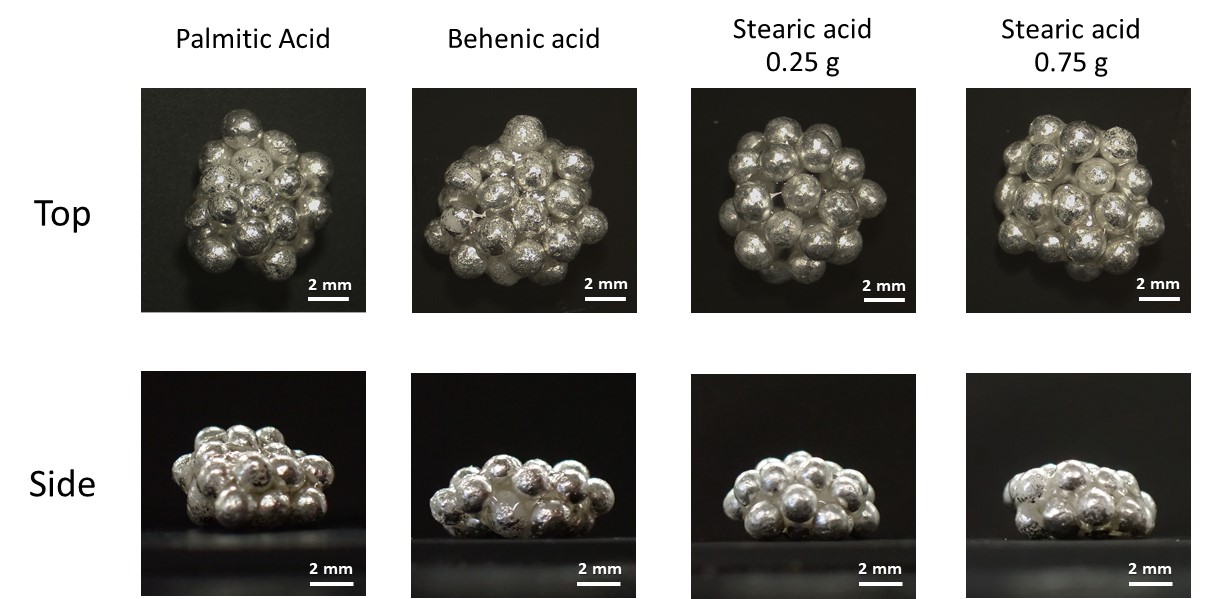


**Figure S3:** Stereomicroscopy images of liquid marbles containing PGL as an inner liquid stabilized with silver dragees whose surfaces were hydrophobized using palmitic acid, behenic acid, and stearic acid. In the case of stearic acid, the surface modification was conducted at SA concentrations of 2.5 g/L and 7.5 g/L: Top and side views.


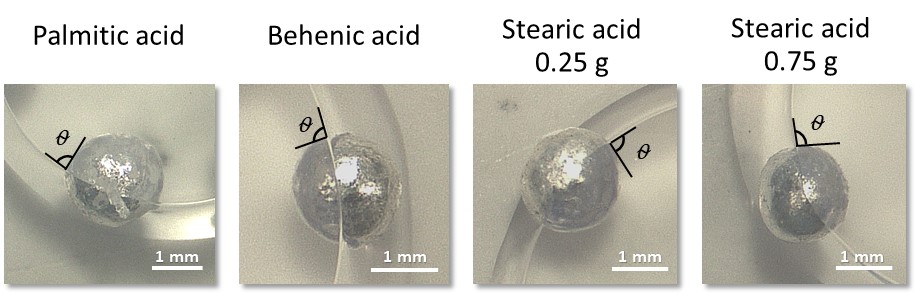


**Figure S4:** Silver dragees, whose surfaces were hydrophobized using palmitic acid, behenic acid, and stearic acid, adsorbed at air-PGL interface. In the case of stearic acid, surface modification was conducted at SA concentrations of 2.5 g/L and 7.5 g/L.


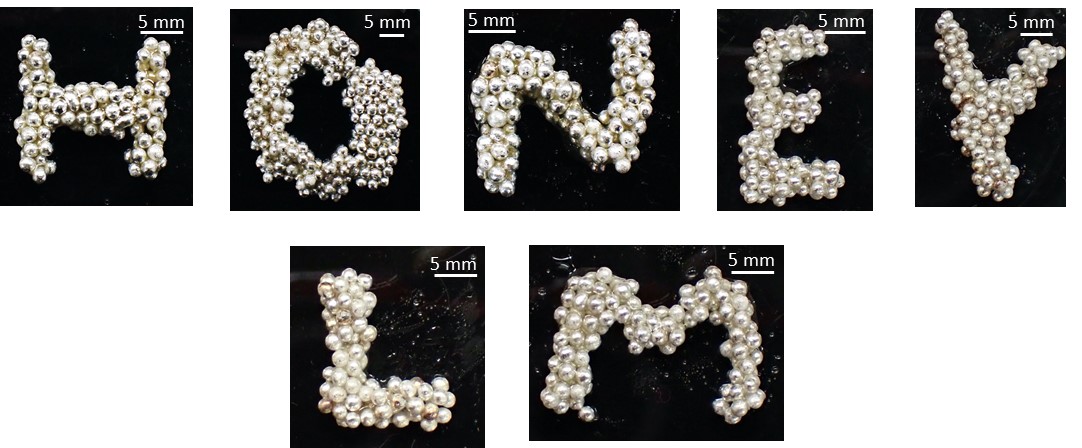


**Figure S5:** Stereomicroscopy images of LMs (honey as inner liquid) stabilized with stearic acid-modified silver dragees shaped as letters.


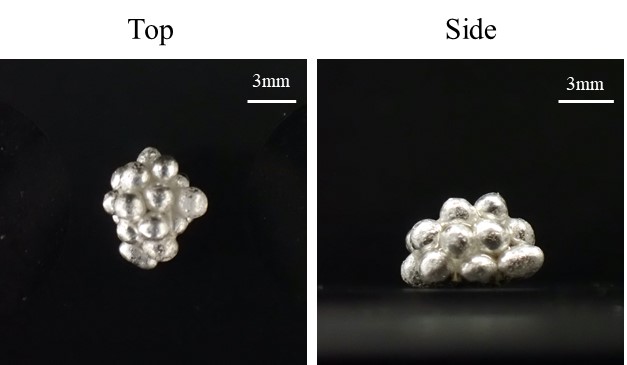
**Figure S6:** Stereomicroscopy images of LM (polyglyceryl monolaurate as inner liquid) stabilized with stearic acid-modified silver dragees: Top and side views.


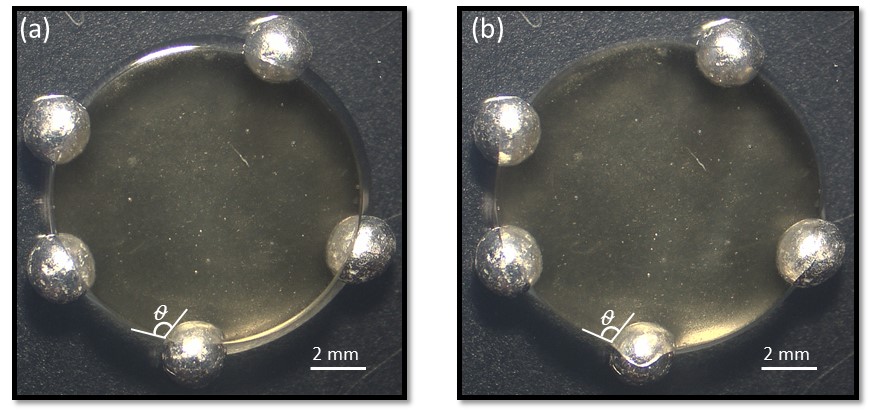


**Figure S7:** Silver dragees adsorbed at air-honey interface: **(a)** before and **(b)** after application of mechanical stress.
